# Risk factors for postcalving intramammary infection with *Mycoplasma* species in first-lactation cows on a conventional dairy farm: A retrospective case control study

**DOI:** 10.3168/jdsc.2025-0915

**Published:** 2026-02-13

**Authors:** Alejandra Zapata-Salazar, Matthias Wieland, Francisco A. Leal Yepes

**Affiliations:** Department of Population Medicine and Diagnostic Sciences, Cornell University, Ithaca, NY 14853

## Abstract

*Mycoplasma* spp. are highly contagious bacteria that cause chronic, subclinical infections in dairy cattle and are intrinsically resistant to many antimicrobials. This retrospective case control study evaluated whether different calfhood and peripartum variables were associated with postcalving intramammary *Mycoplasma* spp. infections. We analyzed data from 475 first-lactation animals from one commercial dairy herd, comprising 95 cases with intramammary infections caused by *Mycoplasma* spp. and 380 culture-negative controls. Data on colostrum Brix%, birth body weight, otitis and pneumonia events, age at first breeding, age at conception, days in the close-up pen, age at calving, days in the fresh cow pen, and calving season were retrospectively extracted from electronic herd records and analyzed using univariable logistic regression models. We found that age at the first otitis event was associated with the occurrence of an intramammary infection with *Mycoplasma* spp. Cows that experienced an otitis event during calfhood, specifically at ≤23 d of age, had greater odds of postcalving intramammary infection with *Mycoplasma* spp. No associations were found for other variables. These results suggest that otitis in early life may represent an important predictor of susceptibility to intramammary *Mycoplasma* spp. infection postcalving and highlight the potential value of calfhood monitoring for mastitis prevention strategies.

## Highlights

• Early-life otitis (≤23 days) was associated with postpartum *Mycoplasma* spp. IMI.
• Cows with early otitis had 2.3× higher odds of *Mycoplasma* spp. infection.
• No other calfhood, reproductive, or management factors were associated.

## Graphical Abstract

**Figure:**
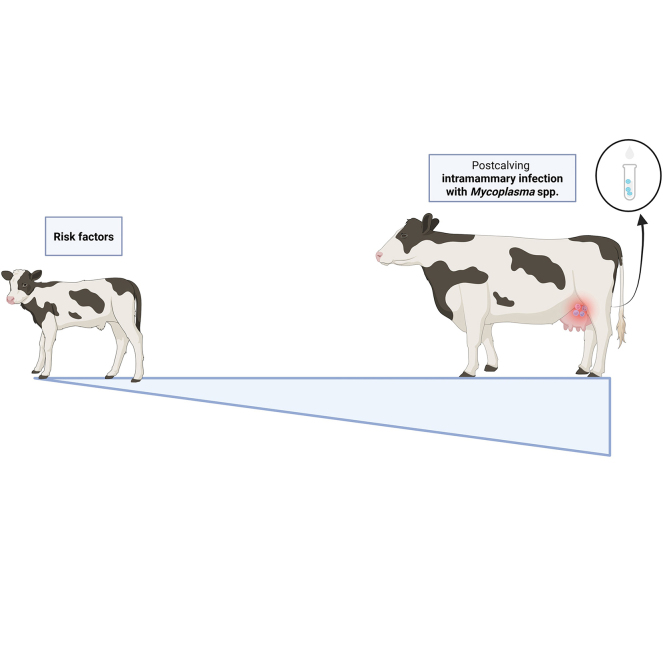
**Summary:** This study investigated whether calfhood and peripartum variables were associated with the development of postcalving intramammary infection (IMI) with *Mycoplasma* spp. in dairy cows. We analyzed data from 475 first-lactation animals using univariable logistic regression models. Cows that experienced their first episode of otitis at ≤23 days of age had 2.3 times higher odds of postcalving IMI with *Mycoplasma* spp. compared with cows that did not experience an otitis event. No significant associations were observed for other calfhood, reproductive, or management variables. These findings suggest that early-life otitis may serve as an important predictor of susceptibility to *Mycoplasma* spp. IMI after calving, underscoring the potential value of calfhood health monitoring as part of mastitis prevention strategies. The figure was created with BioRender.com.

*Mycoplasma* spp. are highly contagious bacteria that have gained increasing attention in the dairy industry due to their intrinsic resistance to antimicrobial therapy. This resistance is primarily attributed to their lack of a cell wall (Maunsell and Donovan, 2009), which renders them naturally resistant to β-lactam antibiotics and several other commonly used antimicrobials (Nicholas and Ayling, 2003). More than 100 species of *Mycoplasma* spp. have been identified (Razin et al., 1998), but *Mycoplasma bovis*, first described in the context of *Mycoplasma* spp. mastitis, remains the primary species of concern in the US dairy cattle industry (Smith et al., 2025).

*Mycoplasma* spp. can adhere firmly to mucosal cell surfaces, with a particular predilection for establishing infections in the respiratory, mammary, or genital systems (Maunsell and Donovan, 2009; Perez-Casal, 2020). Consequently, infections caused by *Mycoplasma* spp. may present with a variety of clinical signs, including those associated with mastitis, conjunctivitis, otitis, arthritis, pneumonia, and urogenital infections (Nicholas and Ayling, 2003; Smith et al., 2025). In the mammary gland, *Mycoplasma* spp. can cause clinical signs of mastitis, but it is most often a subclinical disease, making early diagnosis and cost-effective control strategies challenging (Biddle et al., 2005). Cows infected with *Mycoplasma* spp. might not show any clinical signs even in advanced stages of infection (González and Wilson, 2003). However, substantial economic losses occur whether mastitis is clinical or subclinical, including reduced milk yield and quality and increased culling rates (Gelgie et al., 2022).

The pathogenesis of *Mycoplasma* spp. infections involves complex host–pathogen interactions, including epithelial adherence, cellular internalization, immunomodulation, and persistent colonization without overt clinical signs (Razin et al., 1998; Nicholas and Ayling, 2003). These features enable the organism to evade immune recognition and therapeutic intervention, making control at the herd level particularly challenging.

Despite increasing recognition of *Mycoplasma* spp. as major mastitis pathogens, their epidemiology still remains not fully understood (Maunsell and Donovan, 2009). Chronic asymptomatic infection with intermittent shedding has been recognized as a critical factor in herd-to-herd transmission (Smith et al., 2025). The disease is reported more frequently in large dairy herds, where 2 major risk factors have been identified: (1) the introduction of infected animals and (2) suboptimal milking procedures (Nicholas and Ayling, 2003; Punyapornwithaya et al., 2011; Smith et al., 2025).

Little is known about the animal-level risk factors that may predispose cows to intramammary infections with *Mycoplasma* spp. Therefore, the objective of this retrospective study was to determine whether calfhood and peripartum variables were associated with intramammary infection with *Mycoplasma* spp. postcalving.

This retrospective case control study was conducted on a 4,500-cow dairy herd located in New York State. Because the study relied exclusively on data collected through routine herd management and diagnostic procedures, it was exempt from Institutional Animal Care and Use Committee (IACUC) approval. The herd is part of the client base of Quality Milk Production Services and the Ambulatory and Production Medicine Clinic and was selected based on the owner's willingness to participate in the study. The study population consisted of heifers entering their first lactation that calved between January and December 2023. Individual cow data were extracted from the herd management software, Dairy Comp 305 (Valley Agricultural Software, Tulare, CA).

At calving, a composite aseptic milk sample was obtained from all functional quarters by trained farm personnel, either on the day of calving or between 7 and 13 DIM. All blind quarters were excluded from sampling.

Samples were submitted to the Animal Health Diagnostic Center (Cornell University, Ithaca, NY) and processed for the detection of *Mycoplasma* spp. using standard culture methods in accordance with the guidelines of the National Mastitis Council (NMC, 2017). Briefly, samples were gently mixed by inversion and inoculated with a sterile cotton swab (swab dimensions: 16 × 5 mm; Puritan Medical Products Co., Guilford, ME) onto one half of a modified Hayflick medium (Northeast Laboratory, Winslow, ME). Inoculated plates were incubated at 37°C under conditions of >95% humidity and 5% CO_2_ for 7 d. Plates were examined daily using an illuminated stage stereomicroscope (SZY-ILLB, Olympus, Tokyo, Japan) to identify colonies with morphology consistent with *Mycoplasma* spp.

Cows testing positive for *Mycoplasma* spp. were classified as cases (n = 95). For each case, 4 culture-negative cows were randomly selected as controls (n = 380), yielding a total study population of 475 animals. Control cows were randomly selected using a random number generator in Microsoft Excel. Culture-negative cows served as controls to represent the population at risk and to estimate the association between calfhood and peripartum variables and the likelihood of developing a postcalving intramammary infection. Control cows were matched only by lactation number and sampling period. No a priori sample size calculation was performed; instead, all available animals were enrolled over the course of one year.

The following historical animal-level health and management records were collected: Colostrum Brix (%): Colostrum was harvested immediately after calving and pooled from multiple cows. The pooled colostrum was measured using digital Brix refractometry (Palm Abbe Digital Refractometer, Misco) immediately after collection, before pasteurization, storage, and feeding. The pooled colostrum was filled in 4-L bags (Perfect Udder Bag, Dairytech Inc.), heat treated at 60°C for 60 min in a commercial pasteurizer (Adda Pasteurizer, Gen3, Dairytech Inc.), cooled down with cold water, and frozen at −20°C. Before feeding, colostrum was warmed in a 40°C agitated water bath for 40 min (Matilda Colostrum Warmer, Gen3 Matilda series, Dairytech Inc.). Colostrum was then fed using an esophageal tube feeder. Brix% values of the fed colostrum were recorded individually for each calf by trained farm personnel.

Birth body weight (kg): Calves were weighed within the first 30 min of life, before first colostrum feeding. A Tru-Test S3 Cattle/Livestock Weigh Scale System (Datamars Livestock, New Zealand) was used to obtain the birth body weight.

Age at first otitis (d): The presence of otitis was monitored twice daily by trained farm workers using visual observation. An ear scoring system adapted from the Wisconsin Calf Health Scoring System (McGuirk and Peek, 2014) was used: 0 = normal, 1 = ear flick or head shake, 2 = slight unilateral droop, and 3 = head tilt or bilateral droop. An event was defined as the first recorded deviation from a normal score.

Number of otitis events: The total number of otitis events per calf was counted during the observation period, defined from birth until 175 d of age. A new otitis event was recorded only if at least 7 d had passed since the last appearance of clinical signs.

Age at first pneumonia event (d): Respiratory health was evaluated twice daily by trained farm personnel using a scoring system adapted from the Wisconsin Calf Health Scoring System, which included assessment of nasal discharge (0–3), cough score (0–3), and ocular discharge (0–3). The first day a pneumonia event was recorded was documented as the age at which the first pneumonia event occurred.

Number of pneumonia events: The total number of pneumonia events per calf was recorded during the observation period, defined from birth to 175 d of age. A new pneumonia event was recorded only if clinical signs had been absent for at least 7 d since the previous episode.

Age at first breeding (d): All heifers were bred via artificial insemination following estrus detection using an activity monitoring system, supported by visual observation. First service age was recorded as the age at first breeding. Starting around 370 d of age, heifers that continued to show estrus activity received 2 mL of Synchsure (cloprostenol sodium, Boehringer Ingelheim Animal Health USA Inc., Duluth, GA) at 14-d intervals.

Age at conception (d): Conception was inferred from the continued absence of estrus activity following insemination. Pregnancy was then confirmed in some animals through manual rectal palpation performed by trained farm personnel.

Days in close-up pen: Defined as the number of days spent in the transition pen once animals were ≥250 d of gestation until calving.

Age at calving (d): Calculated as the interval between date of birth and first calving.

Days in fresh cow pen: Defined as the number of days spent in the designated fresh cow pen, corresponding to 0 to 21 d postcalving.

Calving season: Classified according to calendar dates as winter (January 1–March 19 and December 21–31), spring (March 20–June 20), summer (June 21–September 22), and fall (September 23–December 20).

Before conducting statistical analysis, data were examined for missing values and erroneous data. Statistical analyses were conducted using JMP Pro (version 17.0.0, SAS Institute Inc., Cary, NC). To examine the association between the presence or absence of a postcalving intramammary infection with *Mycoplasma* spp. calfhood and periparturient variables, we used univariable logistic regression models. The presence or absence of an intramammary infection with *Mycoplasma* spp. was the dependent variable, and calfhood, health, and management events were the independent variables and tested individually. Associations were tested using the Wald chi-squared test. Due to incomplete records for some animals, the number of observations varied for each variable. After an initial screen through the univariable analyses, we determined the optimal cut-point for the independent variable age at first otitis event by calculating Youden's J statistic of the receiver operating characteristic table, which yielded a cut-point of 23 d. Subsequently, we created a categorical variable for age at first otitis event that included (1) first otitis event at ≤23 d of age, (2) first otitis event at >23 d of age, and (3) no otitis event, facilitating the inclusion of animals that had no record of an otitis event.

Descriptive statistics of animal-level health and management records, together with the final number of animals included in the analysis for each variable, are presented in Table 1. Table 2 shows the odds ratios (**OR**) with corresponding 95% CI from univariable analyses. The most notable result was that age at first otitis event was associated with the occurrence of an intramammary infection with *Mycoplasma* spp. postcalving. Using age at first otitis event as a continuous variable suggested that each additional day reduced the odds (95% CI) of *Mycoplasma* spp. positivity postcalving (OR [95% CI]: 0.04 [0.00–0.44]; *P* = 0.01). Using the categorical variable for age at first otitis event showed that animals experiencing their first otitis event at ≤23 d of age had 2.36 (1.30–4.26; *P* = 0.005) greater odds of *Mycoplasma* spp. positivity postcalving compared with those that did not develop otitis. Similarly, cows that experienced a higher number of otitis events had greater odds of postpartum intramammary infection with *Mycoplasma* spp. (OR [95% CI]: 2.93 [0.91–9.41]), and the likelihood that this difference was due to chance was 7% (*P* = 0.07). In contrast, none of the other calfhood, reproductive, or management variables, including colostrum Brix%, birth weight, pneumonia events, age at breeding or conception, age at calving, days in close-up or fresh pens, and calving season, were associated (*P* ≥ 0.10).

**Table 1:** Comparison of calfhood and peripartum variables (mean ± SD) between *Mycoplasma-*positive and -negative cows^1^

Item^2^	Mycoplasma+		Mycoplasma−	
	n	Mean ± SD	n	Mean ± SD
Colostrum Brix (%)	90	23.8 ± 1.3	352	23.8 ± 1.3
Birth weight (kg)	84	38.9 ± 3.8	305	39.3 ± 4.1
Age at first otitis event (d)	69	28 ± 12.2	248	33.8 ± 16.5
Number of otitis events	95	1.7 ± 1.6	380	1.4 ± 1.5
Age at first pneumonia event (d)	20	43.2 ± 27.9	89	38.9 ± 32.8
Number of pneumonia events	95	0.3 ± 0.6	380	0.3 ± 0.8
Age at first breeding (d)	95	381.7 ± 4.4	380	376.7 ± 26.8
Age at conception (d)	58	388.9 ± 24.8	234	390 ± 38.2
Days in close-up pen	73	22.3 ± 5.5	294	23.1 ± 12.6
Age at calving (d)	69	675 ± 25.5	285	667 ± 47.1
Days in fresh cow pen	85	13.7 ± 3.3	375	13.6 ± 2.7
Calving season, n (%)				
Summer (Jun. 21–Sep. 22)		20 (16.1)		104 (83.9)
Spring (Mar. 20–Jun. 20)		28 (20.3)		110 (79.7)
Fall (Sep. 23–Dec. 20)		26 (24.8)		79 (75.2)
Winter (Jan. 1–Mar. 19 and Dec. 21–31)		21 (19.4)		87 (80.6)

1 Mycoplasma+ indicates samples that tested culture-positive for *Mycoplasma* spp. Mycoplasma− indicates samples that tested culture-negative for *Mycoplasma* spp.

2 Calfhood and peripartum variables evaluated as potential risk factors.

**Table 2:** Calfhood and peripartum variables with the odds ratios, the 95% CI for each of the variables, and *P*-value

Item^1^	Odds ratio (95% CI)	*P*-value^2^
Colostrum Brix (%)	0.94 (0.29–3.01)	0.92
Birth weight (kg)	0.48 (0.08–2.85)	0.42
Age at first otitis event (d)	0.04 (0–0.44)	0.01
Age at first otitis event		0.005
≤23 d	2.36 (1.3–4.26)	
>23 d	1.05 (0.61–1.82)	
No otitis	Referent	
Number of otitis events	2.93 (0.91–9.41)	0.07
Age at first pneumonia event (d)	2.22 (0.12–41.07)	0.59
Number of pneumonia events	0.49 (0.07–3.69)	0.49
Age at first breeding (d)	2.92 (0.81–10.54)	0.1
Age at conception (d)	0.85 (0.19–3.81)	0.84
Days in close-up pen	0.21 (0–45.21)	0.57
Age at calving (d)	5.24 (0.47–58.32)	0.18
Days in fresh cow pen	1.11 (0.21–5.78)	0.9
Calving season		0.45
Summer (Jun. 21–Sep. 22)	0.8 (0.41–1.57)	
Spring (Mar. 20–Jun. 20)	1.05 (0.56–1.98)	
Fall (Sep. 23–Dec. 20)	1.36 (0.71–2.61)	
Winter (Jan. 1–Mar. 19 and Dec. 21–31)	Referent	

1 Calfhood and peripartum variables evaluated as potential risk factors.

2 *P*-value from logistic regression.

This study identified early-life otitis as a risk factor for postcalving intramammary infection with *Mycoplasma* spp. in dairy cows. Specifically, otitis diagnosed at ≤23 d of age was associated with subsequent infection, underscoring the role of calfhood otitis as a potential early marker of susceptibility. Our findings align with previous studies that have shown *Mycoplasma* spp. to be a frequent cause of otitis media in calves, and that such infections may establish a reservoir for later intramammary infections (Biddle et al., 2005; Maunsell et al., 2012). The tendency toward a greater number of otitis events in positive cows further strengthens the hypothesis that recurrent or early-life otitis may be a critical risk factor for the persistence and dissemination of *Mycoplasma* spp. in dairy herds.

Outbreaks of otitis media in North American dairy calves have been predominantly attributed to *M. bovis* (Walz, 1997; Fox, 2012). Experimental evidence supports its role as a primary pathogen of the middle ear. In one study, immunocompetent calves inoculated at 7 to 10 d of age with a field strain of *M. bovis* via milk replacer were consistently colonized in the upper respiratory tract and Eustachian tubes, with 37% developing otitis media within 2 wk postinoculation, and no other pathogens were isolated (Maunsell and Donovan, 2009). Oral inoculation with *M. bovis* has also been shown to lead to colonization of the upper respiratory tract, particularly the tonsils, which can subsequently serve as a silent reservoir for otitis media even when nasal swabs test negative (Maunsell et al., 2012). Moreover, *M. bovis* has been shown to colonize multiple body sites, with identical genotypes detected in milk, urogenital, ocular, and nasal samples from the same cow (Biddle et al., 2005). Although species-level identification was not conducted in this study, a previous diagnostic test and unpublished analysis conducted on the same farm during 2022 to 2023 identified *M. bovis* and *Mycoplasma bovirhinis* in nasal and ear swabs from calves, as well as *M. bovis*, *Mycoplasma canadense*, and *Mycoplasma bovigenitalium* in milk samples from cows. Notably, *M. bovis* was identified across nasal, ear, and milk samples in that earlier analysis, demonstrating its ability to colonize multiple anatomical sites within a single herd. However, because speciation was not performed in the current study, it remains unclear whether the intramammary infections were caused by *M. bovis* or other *Mycoplasma* spp. Thus, establishing a causal link between calfhood otitis and intramammary infection will require future studies that include species-specific identification and strain typing to determine whether the same strains persist from calfhood into lactation.

In one clinical investigation of endemic pneumonia, nearly half of dairy calves were shedding *Mycoplasma* spp. by 5 d of age and more than 90% by 4 wk of age (Stipkovits et al., 2001). In another study, clinical *Mycoplasma* spp. disease in calves was highest between 10 to 15 d (Nicholas and Ayling, 2003). Experimental evidence also indicates that neonatal colonization of the nasopharynx often results in ascending infection of the Eustachian tubes, supporting this as the primary route for middle ear involvement (Maunsell and Donovan, 2009). Although age at the first pneumonia event was not associated with postcalving *Mycoplasma* spp. infection, the association with age at the first otitis event supports the idea that otitis occurring early in calfhood, even without clear pneumonia signs, may be a key indicator of susceptibility to *Mycoplasma* spp. infection.

The predominance of local rather than systemic immune responses in infected calves may further explain why colonized animals often remain asymptomatic while continuing to act as carriers. In practical terms, the results emphasize the importance of monitoring and managing ear infections in young calves as a potential point of intervention to reduce future risk of *Mycoplasma* spp. mastitis. Preventive strategies could include early detection of otitis media, improved calf housing and hygiene, and further investigation into whether targeted treatment or management protocols can reduce the risk of chronic carrier states.

This study has several limitations that should be considered when interpreting the results. The study was conducted on a single dairy farm in New York State, which limits the generalizability of the findings to other management or geographic regions. Because the analysis was retrospective, some variables may have been incompletely recorded. Additionally, pregnancy diagnoses were not routinely performed by a veterinarian or accompanied by laboratory diagnostics, which may have resulted in misclassification within the reproductive records. Species-level identification of *Mycoplasma* spp. isolates was also not performed, preventing confirmation of whether infections were specifically caused by *M. bovis* or by other *Mycoplasma* spp. Finally, blind quarters were not sampled; however, these quarters may harbor *Mycoplasma* spp. and represent a potential source of exposure if not consistently identified or if milking personnel attempt to milk them.

Future research should focus on longitudinal studies that follow calves from birth through lactation, incorporating molecular tools such as PCR and strain typing to confirm whether early-life otitis is directly linked to subsequent intramammary infections. Such approaches will be essential for clarifying the mechanisms underlying the epidemiology of *Mycoplasma* spp., intramammary infection, and for developing effective strategies to mitigate its impact on dairy production.
